# Current position of neuromodulation for bladder pain syndrome/interstitial cystitis

**DOI:** 10.1097/MOU.0000000000001148

**Published:** 2023-11-06

**Authors:** Harry J. Kendall, Julia Schrijvers, John P.F.A. Heesakkers

**Affiliations:** a Department of Urology, Maastricht University Medical Centre; b Maastricht University, Maastricht, The Netherlands

**Keywords:** bladder pain syndrome, interstitial cystitis, neuromodulation, pain, treatment

## Abstract

**Purpose of review:**

Despite established effectiveness in overactive bladder and nonobstructive retention, neuromodulation's application in interstitial cystitis/bladder pain syndrome (IC/BPS) remains a topic of ongoing research. The purpose of this article is to review recent developments in neuromodulation as treatment of IC/BPS offering guidance for healthcare practitioners dealing with IC/BPS cases.

**Recent findings:**

Recent research underlines the promising role of sacral, tibial and pudendal neuromodulation in management of IC/BPS symptoms. Studies reveal encouraging outcomes, particularly in alleviating urgency and frequency symptoms. However, while urgency and frequency symptoms tend to improve, comprehensive pain relief remains a challenge. Percutaneous tibial nerve stimulation (PTNS) and transcutaneous tibial nerve stimulation (TTNS) stand out due to their minimal invasive nature. Existing literature points to the need for larger prospective studies with extended follow-up periods to validate the efficacy and sustainability of neuromodulation.

**Summary:**

Neuromodulation is a promising treatment modality for refractory IC/BPS. Due to the minimal invasive nature, they should be tried before rigorous surgery. However, the limited quantity of available data and the variability in pain relief outcomes necessitate cautious interpretation. The review emphasizes the need for further research.

## INTRODUCTION

Neuromodulation is a promising technique in the treatment of numerous urological and nonurological conditions. Overactive bladder syndrome (OAB), nonobstructive retention (NOR) and faecal incontinence have robust literature regarding neuromodulation and sacral neuromodulation (SNM) has been accepted and implemented in routine care [1]. Practice of neuromodulation is centred in specialised clinics and finds its place in the standard of care when patients prove refractory to other therapies. Targets for modulation of neural pathways are the sacral nerves (S3 or S4) and the tibial nerve, and advancements have been made towards pudendal nerve stimulation.

With that being said for OAB/NOR, literature concerning interstitial cystitis/bladder pain syndrome (IC/BPS) is limited and treatment with neuromodulation is regarded as off-label. IC/BPS has been defined by the International Continence Society (ICS) as persistent or recurrent chronic pelvic pain, pressure or discomfort perceived to be related to the urinary bladder, accompanied by at least one other urinary symptom such as urgent need to void or urinary frequency [2]. The physician should rule out any underlying disease before diagnosis. A hypothesized pathophysiological mechanism of IC/BPS is an altered sensory pathway for nociception [3]. How neuromodulation works is still in the early steps of investigation and currently relies on hypothesis.

In this review, we will discuss the concept of neuromodulation in IC/BPS. We aim to perform a critical analysis of the most recent literature to offer insights into the evolving role of neuromodulation, from SNM to emerging noninvasive techniques. By doing so, we hope to create an overview for healthcare professionals dealing with this treatment modality for IC/BPS in their clinic. 

**Box 1:**
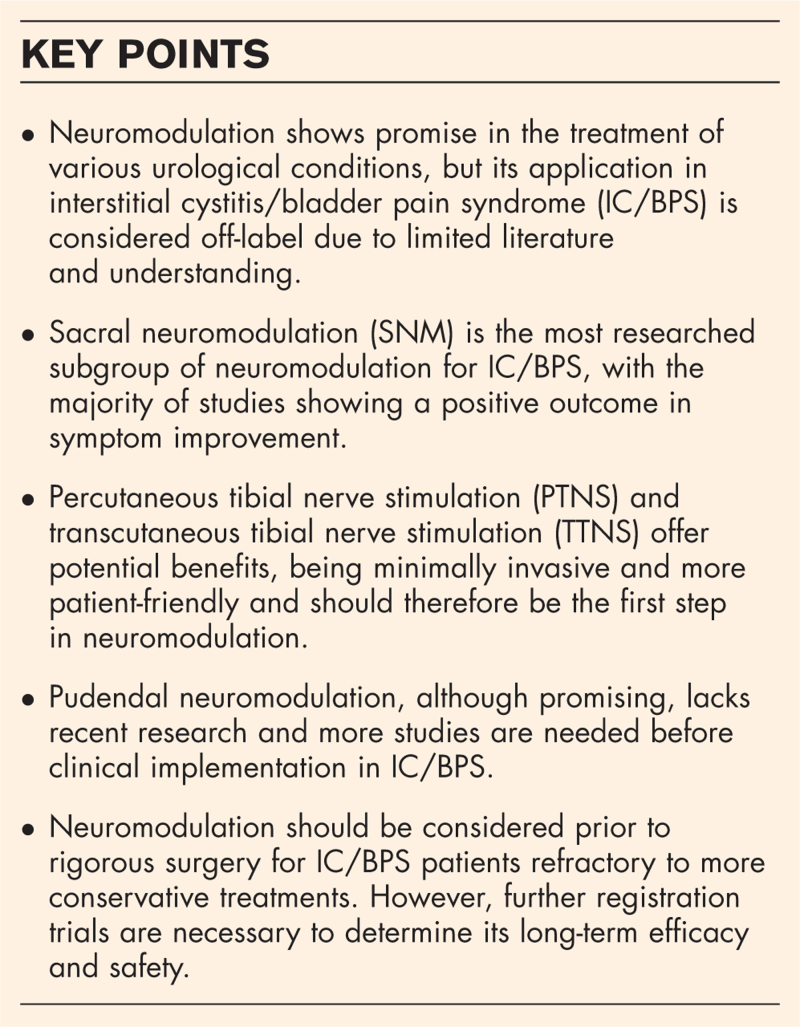
no caption available

## SUMMARY OF CURRENT UNDERSTANDING

The current understanding of the therapeutic mechanism of neuromodulation on IC/BPS is based on afferent upregulation of bladder innervation. The bladder is lined with a glycosaminoglycan (GAG) layer, which has a pivotal role as a protective barrier. We can speculate that disruption of this barrier causes urothelial dysfunction. Specific pathophysiology behind this lies outside the scope of this review and is well described by Akiyama *et al.*[3]. However, urothelial disruption leads to an increased permeability of its barrier function, allowing urinary stimuli access to afferent neurons situated in the subepithelial tissue. This results in modulation of these neurons leading to amplification in the central nervous system. Furthermore, neurogenic inflammation mediated by mast cells could play an important role in altering nociception in sensory pathways.

It is thought that neuromodulation regulates this pain in two ways. The first is through the gate control theory in which pain signals are blocked by administering afferent impulses from nonnociceptive fibres which on their term activate inhibitory interneurons in the dorsal horn of the spinal cord which reduce the activity of nociceptive fibres. Therefore, inhibition of the upregulated nociception in the bladder through neuromodulation theoretically could be an effective treatment modality. Secondly, neuromodulation is hypothesized to induce functional changes in higher brain centres and by doing so controls descending feedback from the brainstem and other regions to the sacral spinal cord. However, the precise mechanism is not yet fully clarified and further research is needed [4–6].

Neuromodulation for the management of IC/BPS encompasses a spectrum of techniques when patients are refractory to conventional therapies. The most established and researched technique is SNM, where the sacral nerves S3 and S4 are targeted. Another option is pudendal neuromodulation (PNM) where an electrode is placed in the vicinity of the pudendal nerve. A more emerging option is using the tibial nerve as target. Percutaneous tibial nerve stimulation (PTNS) and transcutaneous tibial nerve stimulation (TTNS) have made advancements especially for OAB and NOR, and tibial implants have also gained recognition in recent years. Yet, these treatment options still need to find their way into the treatment of IC/BPS.

## SACRAL NEUROMODULATION

The sacral nerves are the most researched target nerve for neuromodulation regarding IC/BPS and lots of retrospective and prospective studies have been published in the last two decades. In SNM, the S3 or S4 nerves are targeted through needle puncture, placement of a lead and implantation of an internal pulse generator (IPG). This surgical procedure is routinely performed in two stages to allow evaluation of the symptom response before implantation of the IPG or explantation of the test system.

A vast amount of retrospective case studies and prospective trials have been published regarding SNM in IC/BPS, often grouped together with other pelvic pain modalities as a chronic pelvic pain syndrome (CPPS). The majority of the results show a positive outcome either by conversion rate from test phase to IPG implant, long-term success rate or symptom improvement in urgency, frequency and pain [7–12]. These studies often have small sample sizes and therefore should be interpreted with caution. A recent study that should be highlighted is a retrospective cohort study by Gandhi *et al.*[13^▪^] in which they describe their 23 years of experience with SNM. They primarily performed percutaneous nerve evaluation (PNE) and described a subpopulation of 200 patients with IC/BPS. PNE was successful in 63,5% of the IC/BPS patients and 59% received an IPG. The explantation rate was 29.7% and did not significantly differ from the OAB group [13^▪^]. Similarly, Hernandez-Hernandez *et al.*[14] in the same year published their experience of 106 patients, including 19 IC/BPS patients in which a 63.15% success rate was found upon testing with PNE or a Tined lead procedure (TLP). They do not appropriately describe the follow-up results in this group; however, they do mention these to be good in the discussion.

This success rate has also recently been highlighted in a meta-analysis by Greig *et al.*[15^▪▪^]. They evaluated a total of 853 patients with CPPS; however, a large number of studies report on IC or BPS. Similar to the previous mentioned studies, the success rate after test phase was 64.3%. An overall meta-analysis of reported pain scores using a visual analogue scale (VAS) showed a mean reduction of -4.64 points and a mean reduction of -4.55 points for IC/BPS patients specifically [15^▪▪^]. SNM is a promising treatment modality for IC/BPS; however, evidence is restricted to small numbers or retrospective analysis. It is included in European Association of Urology (EAU) and American Urology Association (AUA) guidelines as an option when initial treatments have failed but has still to receive Food and Drug Administration (FDA) and European Medicines Agency (EMA) approval [1,16].

## TIBIAL NEUROMODULATION

Another target for neuromodulation is the posterior tibial nerve, which originates from spinal roots L4 through S3. PTNS consists of administering an electric current over a needle electrode in proximity to the posterior tibial nerve and is placed above the medial malleolus. PTNS necessitates weekly outpatient visits and demands high patient adherence. In contrast, TTNS uses electrode pads that are placed above the medial malleolus and is suitable for self-administration, although it requires daily stimulation.

Research regarding PTNS or TTNS is often done in the context of OAB and not in the context of IC/BPS. As a result, studies on PTNS and TTNS for IC/BPS are limited and individual groups are small. In addition, the diagnostic criteria of IC/BPS, disease evaluation scores and the place in line of therapy exhibits substantial variability across studies [17^▪^,^18^]. Currently, implantable tibial neuromodulation devices are being developed. However, these are yet to be introduced into treatment of IC/BPS [19].

A single-arm, dual-centre pilot study by Sudol *et al.* [20] evaluated the effect of PTNS on IC/BPS by looking at Global response assessment (GRA) scale. Although not statistically significant, seven out of 10 individuals reported symptom reduction of some extent after completion of PTNS treatment [20]. This study shares our opinion that GRA might be a better outcome scale for BPS than looking at symptoms such as frequency, urgency or pain alone, given the complexity of the disease.

Kabay *et al.*[21^▪▪^] conducted the only prospective study available, analysing the efficacy of PTNS as a first-line treatment for patients with IC/BPS. They assessed 39 patients, making it the largest cohort study available on the subject. There was a significant improvement in daytime frequency and urgency episodes, with a mean reduction of 3.8 voids and 4.7 episodes, respectively. Symptom scores showed statistical improvement in pain severity, symptom index and problem index as assessed by Interstitial Cystitis Symptom Index (ICSI), Interstitial Cystitis Problem Index (ICPI) and Visual Analog Scale (VAS) scores [21^▪▪^].

A recently published retrospective chart study by Abdalla *et al.* [22] further provides valuable insights into the potential benefits of PTNS in the treatment of refractory IC/BPS. The study included a total of 34 patients, with 27 of them completing the PTNS treatment. The results demonstrated a statistically significant improvement in urgency severity rates and reduced nocturnal urinary frequency among the treated patients. However, improvements in daytime void frequency and in pain domain were observed, but not statistically significant. Furthermore, the study highlighted that 48.1% of patients successfully transitioned to PTNS maintenance therapy [22].

Alkis *et al.*[17^▪^] is one of the only studies reporting the use of TTNS in the treatment of IC/BPS. TTNS demonstrated statistically significant improvement in median daytime frequency, nocturia, VAS-score and ICSI-score and voiding volume [17^▪^]. Importantly, none of the mentioned studies reported any adverse effects associated with PTNS or TTNS. We highlight TTNS for its ease of application and patient-friendly characteristics, making it an appealing treatment option in the management of IC/BPS.

## PUDENDAL NEUROMODULATION

As the pudendal nerve originates from the S2 through S4 nerve root, this also forms a target nerve for neuromodulation. The pudendal nerve has a great amount of afferent nerve fibres and plays a role in innervation of the pelvic floor alongside the external urethral and anal sphincter [6].

No further research has been published towards PNM for the treatment of IC/BPS since 2010 and two mentionable studies were published around this time. The first prospective, randomized crossover trial between sacral and pudendal nerve stimulation for interstitial cystitis was conducted by Peters *et al.* [23] with a follow up of 6 months. Twenty-two patients received SNM and PNM during one surgery, both using a posterior approach and tested each lead blindly for 7 days. There was a 77% conversion rate and symptom improvement rate was significantly higher for PNM compared to SNM. Thirteen of 17 patients chose the pudendal lead for definitive implant. They found a significant improvement in urgency and frequency but not in pain. Follow up results should be interpreted with caution due to the study not being powered towards this and a large difference in number between the sacral and pudendal group [23]. A retrospective cohort analysing chronic PNM was published by the same leading author three years later. They report on a subgroup of 42 IC/BPS patients of whom 61.9% received an IPG. In a follow-up group, of these patients a significant reduction in ICSI and ICPI scores was found [24].

## TRANSCUTANEOUS ELECTRICAL NERVE STIMULATION

As described above, TTNS can be used as a treatment modality for IC/BPS. This technique can also be used in the pelvic and abdominal region in which it is called transcutaneous electrical nerve stimulation (TENS). A parallel randomized controlled trial by Hacad *et al.* (2022) revealed that TENS using a frequency of 100 Hz in combination with conventional therapy (biofeedback and manual therapy) exhibited significant improvement in urinary symptoms compared to conventional therapy alone [25]. Regarding chronic pelvic pain (CPP), Sharma *et al.*[26] performed a prospective experimental study towards TENS wherein they evaluated a sham control group against three groups which received stimulation on different frequencies. Their results show a significant reduction of pelvic pain upon treatment with TENS with the highest reduction rate being found when stimulating with high frequencies between 75 and 100 Hz [26].

## EXPERT PERSPECTIVE

The cause and pathophysiology of IC/BPS remains unknown, leading to an undetermined treatment strategy. A combination of behavioural therapy and lifestyle changes, pelvic physiotherapy and different pharmacological agents are included in the current approach. Developments are taking place regarding the role of neuromodulation for the treatment of IC/BPS. When all other treatments have failed, a selected group of patients can benefit from neuromodulation, which is minimal invasive and easily reversible. In our opinion, it should be tried before more rigorous surgery is performed. The first step of neuromodulation, should be the least invasive, being TTNS or PTNS. Although patient adherence is of great importance, we highlight the ease of application and patient friendly characteristics, making it an appealing treatment option. More importantly, in the studies mentioned before, no adverse effects have been reported for both PTNS and TTNS. If PTNS or TTNS was unsuccessful or efficacy was lost, SNM should be considered next. Literature points towards a positive effect of SNM by a high conversion rate from test phase to IPG implant. However, it is important to note that the majority of studies on neuromodulation for IC/BPS are based on small sample sized. Although there is some promising, yet outdates evidence available, pudendal nerve stimulation should be investigated further before being implemented in clinical treatment. In the highlighted studies, urgency and frequency complaints often diminish, but pain scores do not always prove to be significantly different. Nevertheless, patients often report a general improvement which we justify by the range of symptoms that can be found in IC/BPS. Therefore, patient counselling on what to expect is of high importance before any intervention occurs. Another reason for careful patient selection are the costs of implant surgery. With the implanted devices often being explanted due to infection, pain or loss of efficacy this should also be taken into consideration.

## FUTURE DIRECTIONS

Neuromodulation is included in the EAU [1] and AUA [16] guidelines as an option for CPPS which encompasses IC/BPS when initial treatment has failed, further registration trials should be conducted to also receive FDA and EMA approval for neuromodulation in IC/BPS. Due to IC/BPS symptoms having an overlap with those of OAB, patients often fit the criteria to receive neuromodulation. However, upon recognition of IC/BPS being treatable by neuromodulation, setting up trials will be easier and advancement in this field can be made. We searched clinicaltrials.gov for neuromodulation in IC/BPS and identified two interesting PTNS trials. Both have been completed but have yet to be published. In the first study, female individuals are randomized between PTNS and Sham for IC/BPS. The second study is a small pilot study with 12-week follow-up.

## CONCLUSION

We conclude that neuromodulation in general is a promising technique in the treatment of IC/BPS that should be considered in patients refractory to more conservative treatment. Due to the minimal invasive nature, we advise tibial neuromodulation in the form of PTNS or TTNS and TENS to be tried prior to sacral or PNM. Success rates seem to be slightly lower than for OAB and results on long-term follow-up are lacking. The main reason for improvement seems to be based on urgency and frequency symptoms improving, however, subjective pain scores do tend to decline alongside other symptoms. Nevertheless, data is of low quantity and should be interpreted with caution. More large prospective studies are required to assess efficacy with extended periods of follow-up.

## Acknowledgements

*None.*

### Financial support and sponsorship

*None.*

### Conflicts of interest

*J.H. and H.K. have grants for studies for Medtronic, Saluda, Bluewind, Neuspera and Innocon.*

## REFERENCES AND RECOMMENDED READING

Papers of particular interest, published within the annual period of review, have been highlighted as:

▪ of special interest
▪▪ of outstanding interest
